# A miniaturized phenotyping platform for individual plants using multi-view stereo 3D reconstruction

**DOI:** 10.3389/fpls.2022.897746

**Published:** 2022-08-08

**Authors:** Sheng Wu, Weiliang Wen, Wenbo Gou, Xianju Lu, Wenqi Zhang, Chenxi Zheng, Zhiwei Xiang, Liping Chen, Xinyu Guo

**Affiliations:** ^1^Information Technology Research Center, Beijing Academy of Agriculture and Forestry Sciences, Beijing, China; ^2^Beijing Key Lab of Digital Plant, National Engineering Research Center for Information Technology in Agriculture, Beijing, China; ^3^Intelligent Equipment Research Center, Beijing Academy of Agriculture and Forestry Sciences, Beijing, China; ^4^College of Agricultural Engineering, Jiangsu University, Zhenjiang, China

**Keywords:** MVS-Pheno, multi-view stereo reconstruction, three-dimensional point cloud, phenotyping platform, wheat

## Abstract

Plant phenotyping is essential in plant breeding and management. High-throughput data acquisition and automatic phenotypes extraction are common concerns in plant phenotyping. Despite the development of phenotyping platforms and the realization of high-throughput three-dimensional (3D) data acquisition in tall plants, such as maize, handling small-size plants with complex structural features remains a challenge. This study developed a miniaturized shoot phenotyping platform MVS-Pheno V2 focusing on low plant shoots. The platform is an improvement of MVS-Pheno V1 and was developed based on multi-view stereo 3D reconstruction. It has the following four components: Hardware, wireless communication and control, data acquisition system, and data processing system. The hardware sets the rotation on top of the platform, separating plants to be static while rotating. A novel local network was established to realize wireless communication and control; thus, preventing cable twining. The data processing system was developed to calibrate point clouds and extract phenotypes, including plant height, leaf area, projected area, shoot volume, and compactness. This study used three cultivars of wheat shoots at four growth stages to test the performance of the platform. The mean absolute percentage error of point cloud calibration was 0.585%. The squared correlation coefficient *R*^2^ was 0.9991, 0.9949, and 0.9693 for plant height, leaf length, and leaf width, respectively. The root mean squared error (RMSE) was 0.6996, 0.4531, and 0.1174 cm for plant height, leaf length, and leaf width. The MVS-Pheno V2 platform provides an alternative solution for high-throughput phenotyping of low individual plants and is especially suitable for shoot architecture-related plant breeding and management studies.

## Introduction

Multi-omics research is currently a hotspot in plant science, as shown by the emerging genomics–phenomics studies (Yang et al., 2013). Although a rapid advancement in genomics has achieved a high-throughput and predictable cost of gene sequencing, phenomics has become the bottleneck of plant multi-omics research (Furbank and Tester, 2011). Plant phenomics is based on obtaining high-quality and reproducible phenotypic traits in a high-throughput manner to quantitatively analyze the genotype and environment interactions and their effects on yield, quality, stress resistance, and other relevant traits (Jin et al., 2020). Satisfactory phenotypic platforms can promote the development of plant phenomics (Jin et al., 2020), enhancing the breeding process and providing data for accurate agricultural management decisions (Shakoor et al., 2017).

Plant phenotyping can be divided into several scales from macro to micro, including regional, population, individual plant, organ, and micro scales. However, ensuring the accuracy and throughput of plant phenotyping at different scales remains a challenge (Zhao et al., 2019; Jin et al., 2021). Many studies have conducted high-throughput phenotyping at the individual plant and organ scales due to the high demand for morphological and structural phenotyping in shoot architecture crop breeding and decision-making in agriculture management (Wu et al., 2019; Xiang et al., 2019; Xiao et al., 2021). Moreover, LiDAR (Wu et al., 2019), depth camera (McCormick et al., 2016; Teng et al., 2021), and high-resolution cameras (Bernotas et al., 2019; Li et al., 2020) are the common data acquisition sensors for morphological and structural phenotyping at individual plant and organ scales. For instance, multi-view stereo (MVS) image three-dimensional (3D) reconstruction is widely used to study morphological and structural phenotyping. It is considered to be the optimal solution to build a high-throughput and low-cost phenotyping platform for individual plants. The previous studies have shown that the phenotypes retrieved from MVS reconstruction can match the accuracy of LiDAR and reconstruct a high-quality 3D point cloud with vertex colors (Wang et al., 2019). How to quickly obtain high-quality plant multi-view images is the core of high-throughput acquisition of MVS-based phenotyping. Based on the relative motion relationship between the target plant and the camera sensor, plant multi-view image acquisition technology can be classified into two modes, i.e., “plant to camera” and “camera to plant” (Fiorani and Schurr, 2013).

In the “plant to camera” mode, the target plant is put on a rotating turntable, rotated, then cameras are fixed to obtain multi-view images of the plant (Zhu et al., 2020b). The number of cameras installed depends on the size of the plant and camera angle of view (Nguyen et al., 2016; Zhang et al., 2016; Gibbs et al., 2018). The first step involves removing the background and retaining only the plant in MVS images. Black or high-contrast colors with green are usually used as the background (Lu et al., 2020). However, tall plants or plants with flexible organs are easily shaken when rotating on the turntable, resulting in poor reconstruction point clouds, such as fuzzy edges of plant leaves and thicker stems than the actual ones. Therefore, it is mostly used to reconstruct 3D models of small plants, seedlings, or plant organs (He et al., 2017; Liu et al., 2017; Syngelaki et al., 2018).

In the “camera to plant” mode, the target plant is maintained at a static position, then one or more cameras are rotated around the plant to obtain multi-view images. The number of cameras required depends on the plant size. The overlap of adjacent images on the same layer should be more than 60%, and that of adjacent images on different layers more than 50%. Multi-view images for low plants such as pepper, eggplant, and cucumber, can be manually obtained without automating the acquisition process (Rose et al., 2015; Hui et al., 2018). However, a manual acquisition cannot ensure the uniformity of image acquisition location and overlap requirements of images; thus, not suitable for large-scale phenotyping applications. As a result, researchers have mounted cameras on flexible rocker arms to achieve automatic data acquisition, improving the automation level of data acquisition and meeting the needs of high-throughput data acquisition (Cao et al., 2019). A motor was needed to drive the rotating arm for tall plants, such as maize, and cameras were installed on the rotating arm to acquire shoot images while rotating (Wu et al., 2020). Since the target plants must remain static while the cameras take images, a higher reconstruction accuracy is obtained using this mode than the “plant to camera” mode. The “camera-to-plant” mode also supports *in situ*, continuous, and non-destructive measurement of individual plants or populations. The operators can directly take images around plants in the field (Walter et al., 2018; Xiao et al., 2020; Zhu et al., 2020a) or install the camera on the phenotypic vehicle (Sun et al., 2020) or mount it on an unmanned aerial vehicle (Zermas et al., 2019; Di Gennaro and Matese, 2020; Wang et al., 2021). Researchers compared the 3D reconstruction effects of the two MVS modes under the same image acquisition environment and they showed that “camera to plant” mode has higher accuracy and robustness than “plant to camera” mode (Gao et al., 2021).

Presently, “camera to plant” mode is used for 3D point cloud reconstruction and phenotypes extraction of plants with relatively simple morphological structure of single stem and large leaves, such as maize. A phenotyping platform MVS-Pheno V1 was developed for maize shoots using “camera to plant” mode (Wu et al., 2020). This platform allows for automatic multi-view images acquisition and structural phenotypes extraction approaches are integrated into the data processing system. However, the platform takes large space to deploy, and the turntable causes the target plant to tremble slightly while rotating. During image acquisition, light and airflow control are not taken into account. Despite of these shortcomings, MVS-Pheno was an advanced platform for automatic obtaining multi-view images of plants. None literature was found to improve MVS-Pheno platform.

For plants with many tillers, slender leaves, and serious shielding, such as wheat and rice, the reconstructed 3D point cloud using multi-view images is usually incomplete, with missing points on leaves and tips (Pound et al., 2014; Duan et al., 2016). Plants with complex morphological structures require enhanced multi-view image data acquisition and reconstruction.

The limitations and challenges in the previous MVS-based plant phenotyping studies can be summarized as follows: (1) Manual acquisition of multi-view images for plants is difficult to ensure the overlapping of incident images and achieve high-throughput acquisition. Automatic acquisition devices that require limited spaces to deploy are urgently needed. (2) Images acquired in open environment conditions are greatly affected by lights and winds, resulting in unsatisfactory reconstruction point clouds. Controlled imaging condition is favorite for such devices. (3) The rotation in “camera to plant” mode is easy to produce cable surrounded, resulting in the inability of continuous and automatic acquisition. (4) Point clouds obtained through multi-view image reconstruction are scaled with different size, automatic scale calibration has to be resolved for further batched phenotypes extraction. (5) The structure and detailed morphology of wheat are more complicated than maize plants, which puts forward higher requirements of acquired images for point cloud reconstruction.

Herein, a miniaturized shoot phenotyping platform MVS-Pheno V2 was developed for low plants, by optimizing the previous platform MVS-Pheno V1 (Wu et al., 2020). Besides, an automatic data acquisition and data processing pipeline for wheat has been constructed and evaluated. Therefore, MVS-Pheno V2 can provide a high-throughput and cost-effective solution for small-scale plant phenotyping of individual plants and organs.

## Materials

This study used three winter wheat cultivars, FengKang13 (FK), JiMai44 (JM), and XiNong979 (XN), with different shoot architectures. The experiment was performed at the experimental field of *Beijing Academy of Agricultural and Forestry Sciences* (39°56′N, 116°16′E). Wheat was planted on 25 September 2020 with a density of 66,666 plants/ha and a row spacing of 16 cm. Sufficient water and fertilizer were supplied during the entire growth period. The field shoots were sampled from the beginning of returning to green stage to booting stage on 23 March 2021, 2 April 2021, 13 April 2021, and 19 April 2021 (three replicates per cultivar). Wheat shoots with different tiller numbers were selected during sampling to test the performance of the platform and verify the adaptability of data processing approaches to different tiller densities. The detailed measured tiller numbers of each sampled shoot are shown in Table 1. The sampled shoots were quickly transplanted to pots, and appropriate water was added to the pots to prevent leaf wilting. The MVS-Pheno V2 platform was used to obtain multi-view images of the shoots. After multi-view image acquisition, a 3D digitizer Fastrak (Polhemus, Colchester, VT, USA) was used to acquire morphological feature points of leaves of several shoots and tillers (Wang et al., 2019), which was used for data verification to evaluate point cloud reconstruction accuracy of MVS-Pheno V2 platform. Moreover, the multi-view images of pepper and eggplant shoots at the seeding stage, tomato, beet, lettuce, chamomile shoots, maize tassel and ears, and roots were acquired using the platform to test its performance on other plants and plant organs.

**Table 1:** Tiller numbers of each sampled wheat shoot in four growth stages. Each cultivar involves three sample replicates.

**Cultivar**	**FK**			**JM**			**XN**		
**Sample ID**	**1**	**2**	**3**	**1**	**2**	**3**	**1**	**2**	**3**
2021/03/23	6	4	6	5	8	10	4	4	2
2021/04/02	6	6	8	7	8	10	4	6	4
2021/04/13	7	8	6	5	7	9	4	7	4
2021/04/19	8	4	3	8	3	5	4	8	7

## Methods

### Platform overview

**Figure 1:**
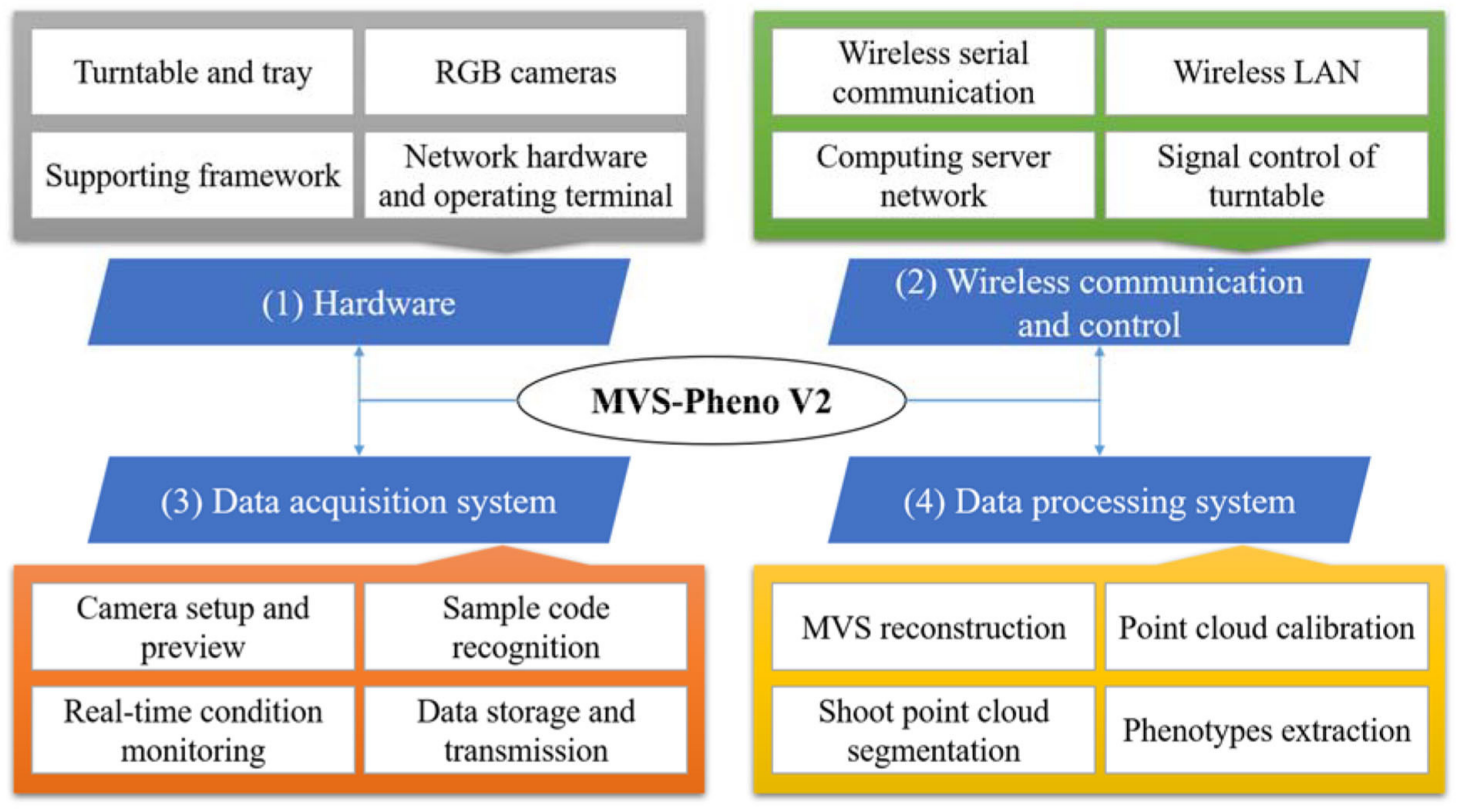
Composition of MVS-Pheno V2 platform.

The MVS-Pheno V2 platform has four components: hardware, wireless communication and control, data acquisition system, and data processing system (Figure 1). (1) The hardware comprised a supporting framework, driving motor, camera sensors, and computers. (2) The wireless communication and turntable control among controllers and operating computers were established by building a local wireless network to reduce the number of physical transmission cables and improve the ease of use and stability of the equipment. (3) The data acquisition system was used to realize automatic data acquisition and provide real-time working conditions to monitor the data acquisition process. (4) The pipeline data processing system was deployed on the server to realize 3D point cloud reconstruction and phenotypes extraction from the acquired multi-view images.

### Hardware

**Figure 2:**
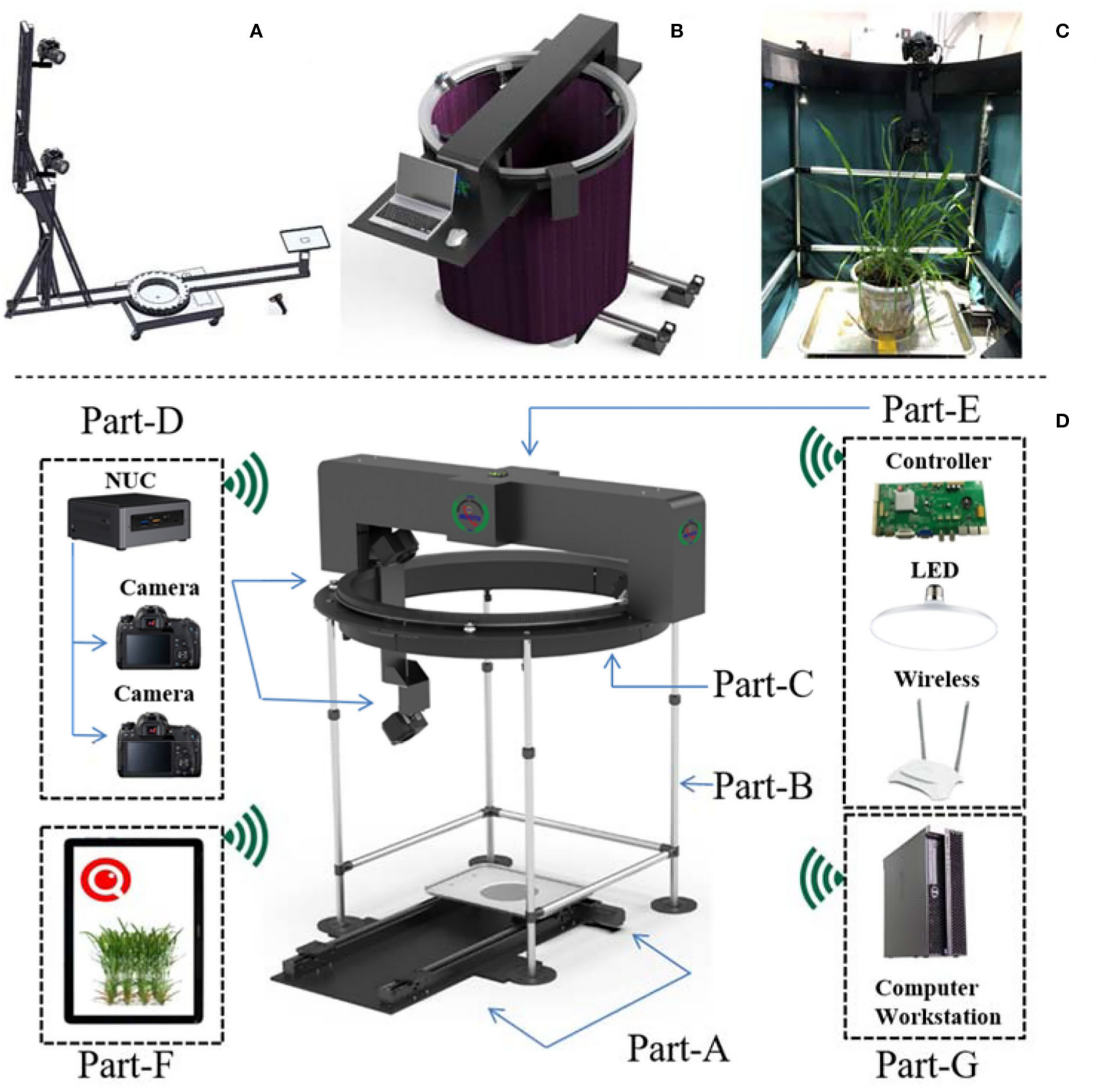
Hardware of MVS-Pheno V2. **(A)** Structural composition of MVS-Pheno V1. **(B)** Overall appearance of MVS-Pheno V2. **(C)** The actual scene inside the platform when acquiring multi-view images of wheat shoots. **(D)** Detailed hardware composition of MVS-Pheno V2 platform.

The MVS-Pheno V1 platform (Wu et al., 2020) was designed to automatically acquire multi-view images of tall plants; thus, its hardware is relatively tall and large (Figure 2A). After a target plant is placed in the center of the platform, a supporting arm with several cameras rotates around the target plant to obtain images.

The MVS-Pheno V2 is an improvement of the MVS-Pheno V1, with the most important improvement being the mode of driving. Since the V2 platform is designed for low plants, the V2 is relatively smaller than the V1, and the driving system is set above the target plant (Figures 2B,C), while the V1 platform drives the supporting arm and cameras near the ground (Figure 2A). The hardware of the V2 platform has seven units, including the shoot transportation unit, supporting unit, rotating unit, data acquisition unit, top unit, operating terminal, and computing server unit (Figure 2D, Supplementary Video 1).

The transportation unit (Figure 2D, Part-A) has a parallel rail, a stepping motor, and a tray for laying plants and transports the target plant to the center of the hardware. A marker plate should be laid near target plant for further point cloud calibration, while not be too close to the target plant during image acquisition to avoid sheltering by leaves. The supporting unit (Figure 2D, Part-B) has four retractable support columns and a cloth fence to construct a wind-shield module (Figure 2B). It supports the upper turntable and blocks additional lights and airflow. The adjustment height of the support columns is 100–160 cm. An opaque and non-reflective double-layer flannelette was adopted as the wind-shield. The rotating unit (Figure 2D, Part-C) has a turntable with the ring gear, a stepping motor, and a position switch. It is placed on the support unit to drive the acquisition unit to rotate around the target plant. The turntable has an inner radius of 60 cm and is customized based on specific requirements. The data acquisition unit (Figure 2D, Part-D) consists of a vertical arm, a data acquisition computer, and one or more cameras. The upper part of the vertical arm is mounted on the turntable and rotates with the turntable. The vertical arm has a height of 100 cm, and at most, three cameras can be installed on the arm. Canon77D cameras with 24-mm half-frame fixed focus lenses are used. An acquisition computer is installed at the bottom of the vertical arm to connect easily to the cameras through data cables. Herein, NUC, a mini-computer produced by Intel, was used. The computer is small and light (size: 11 × 11 × 5 cm, weight: 300 g) and can be mounted on the vertical arm to rotate synchronously with the turntable. The top unit (Figure 2D, Part-E) has a light supplement, electric control, and network modules. The LED light sources are installed in two layers. The upper layer four light sources (10-mm diameter, 20 W white light source) are in the cross-structure of the top unit, and the second layer four light sources (6-mm diameter, 16-W white light source) are on the support columns of the supporting unit. The light sources are inclined downward to avoid the backlight caused by being opposite to the cameras. A router is installed on the upper part (providing LAN network for NUC and operating terminal). This unit also has a motor control circuit board of the platform. The operating terminal (Figure 2D, Part-F) can be wirelessly separated from the platform for the effective operation of the equipment. A mobile phone, iPad, or laptop can also serve to setup the operation terminal (Figures 2B,D, Part-F). The computing server unit (Figure 2D, Part-G) is optional and is connected with the NUC through the network to receive the acquired multi-view images for phenotypes estimation.

### Wireless communication and control

**Figure 3:**
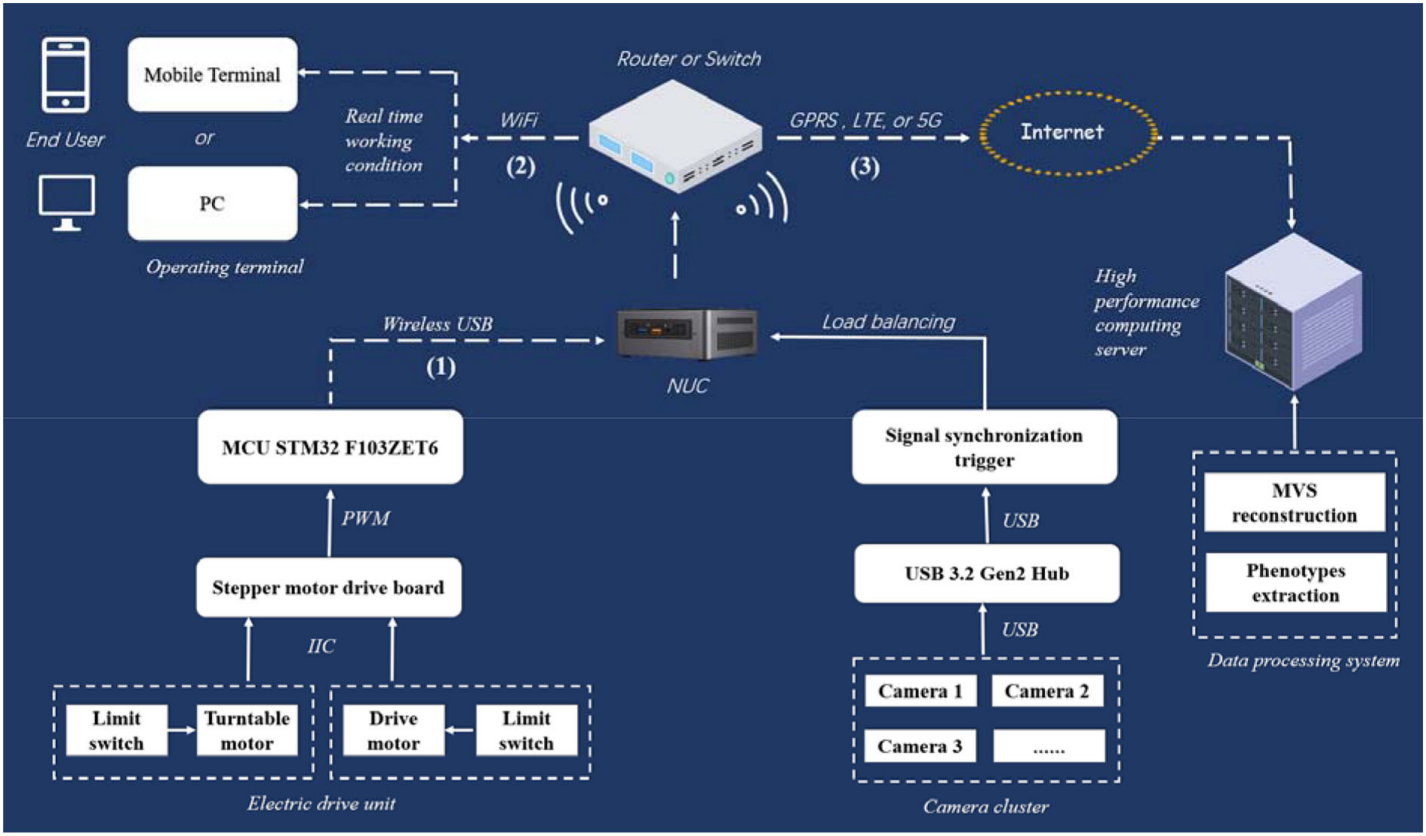
Physical circuit diagram of network communication and signal control in MVS-Pheno V2 platform.

Routing strategy for signal and data transmission is complex in the MVS-Pheno V1 platform. Therefore, a wireless communication network among computers, controllers, and operation terminals established in MVS-Pheno V2 prevents winding among cables during turntable rotation. A detailed physical circuit diagram of network communication and signal control of the platform is shown in Figure 3. Wireless communication has three parts, i.e., WiFi serial communication, LAN, and network channel as discussed in the following: (1) The WiFi serial communication (USB to wireless serial communication, XMS, China) is used to realize the wireless connection between the electronic drive unit and NUC. (2) A LAN is located between the NUC and the operating terminal through a wireless router (wireless Gigabit router, Huawei, China), and facilitates the operating terminals; thus, remotely controlling the NUC. (3) A network channel is established between NUC and a high-performance computing server to transmit the acquired multi-view images in a fixed time.

The platform realizes automatic control through switches. Limit switches are set at the end of the transportation unit and side of the turntable, to detect the motion state of the plant on the rail in the transportation unit and the rotating state of the turntable in rotating unit in real-time. When triggered, the limit switch of the transportation unit sends an electrical signal to the control board to start rotation of the turntable. It triggers the NUC to start image acquisition. The limit switch of the turntable sends an electrical signal to inform the control board to stop the rotation of the turntable and inform the NUC to finish data acquisition when triggered. Moreover, the turntable alternately rotates forward and reverse to prevent the power supply cable twining on the rotating arm (the NUC and cameras provide a 220-V power supply). The load balancing and signal synchronous triggered acquisition mechanism are used to achieve synchronous acquisition and data transmission of multiple cameras.

### Data acquisition system

The data acquisition system on the NUC acquires data automatically, allowing for real-time monitoring of working conditions. The system adopts a linear working flow and involves five steps, sample preparation, labeling, sample incoming, data acquisition, and sample outgoing as detailed in the following: (1) *Sample preparation*. Target plant shoots are planted or transplanted in pots, then placed on the tray of the transportation unit one after another. (2) *Labeling*. Each shoot has an identifier (ID). The ID can be entered using the keyboard or scanned using a code scanning gun on a prepared bar code. A corresponding data storage directory is then established. (3) *Sample incoming*. The transportation unit transports each plant to the end of the rail (the central position of the device) at a constant speed. (4) *Data acquisition*. The limit switch sends a command to the NUC. Then the acquisition system sends a rotation command to the turntable after a delay of 3 s to synchronously drive the camera to acquire multi-view images. The delay prevents the vibration of plants once transportation stops. The turntable rotates at a constant speed, and the camera takes images at same time intervals. The limit switch sends a command to the NUC, then the turntable stops rotating, and the camera stops taking images after the turntable rotates one cycle. (5) *Sample outgoing*. The transportation unit drives the plant out of the equipment. The multi-view image acquisition cycle is continuous, and each cycle usually takes 90 s. Around 28 images cloud be taken in each cycle with 60% overlapping between consecutive images. The data acquisition system was developed using the QT platform (a cross-platform C++ graphical user interface application development framework developed by QT company) and was tested on a workstation with a Win10 operating system (3.2-GHz processor and 8 GB memory).

### Data processing system

The data processing system mainly includes MVS-based 3D point cloud reconstruction, point cloud calibration, shoot point cloud segmentation, and phenotype extraction. The system was developed using OpenGL and PCL (Point Cloud Library) and was tested on a workstation with a Win10 operating system (3.2 GHz processor and 64 GB memory).

#### MVS-based 3D point cloud reconstruction

**Figure 4:**
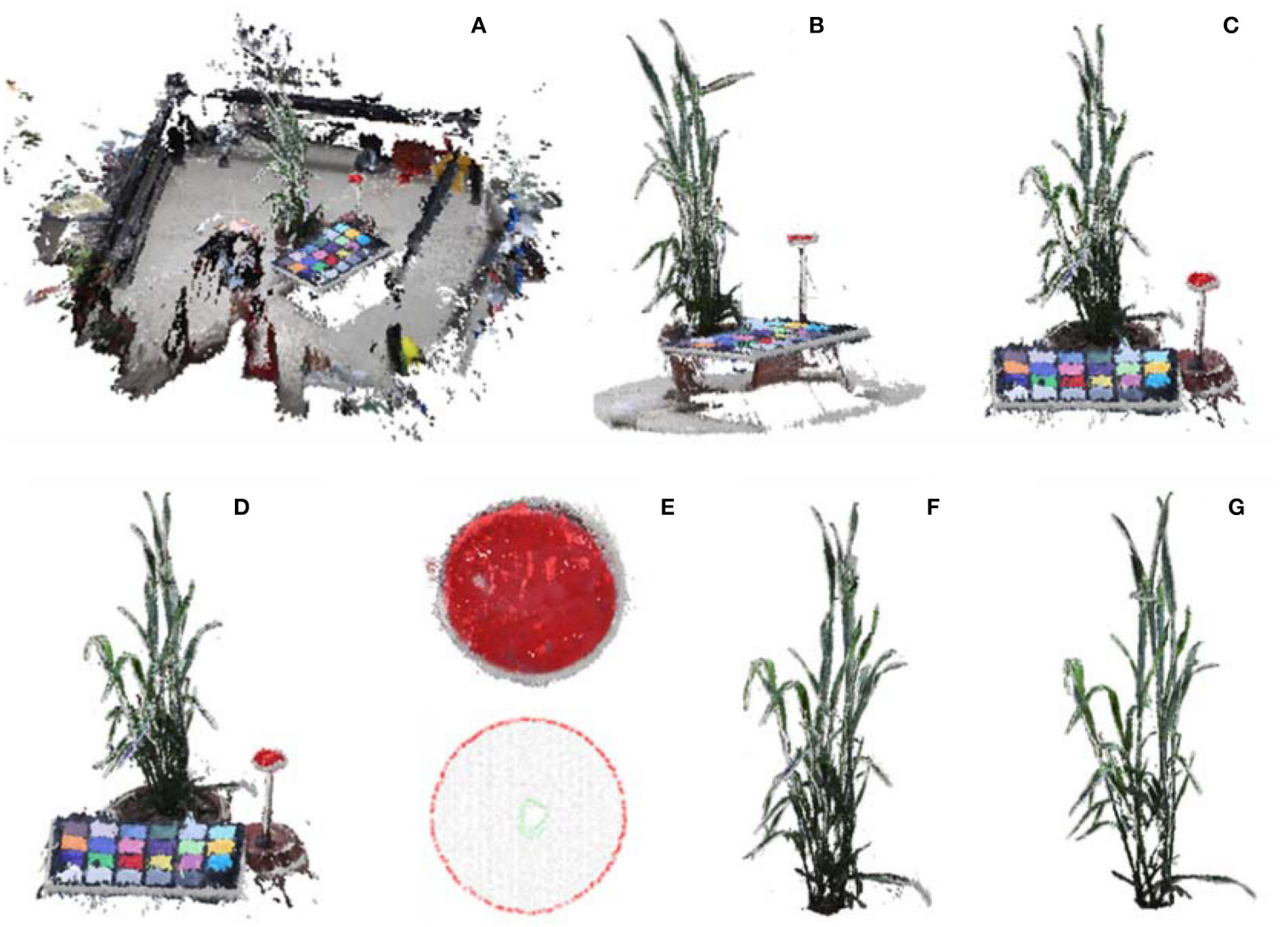
Point cloud calibration and shoot segmentation. **(A)** Reconstructed initial point cloud of scene inside MVS-Pheno V2 platform using the acquired multi-view images. **(B)** Point cloud cropping via cylinder. **(C)** Ground point cloud removal. **(D)** Down sampling of shoot point cloud. **(E)** Marker plate extraction and radius estimation for point cloud calibration. **(F)** Shoot point cloud segmentation. **(G)** Shoot point cloud denoising.

For data processing, the acquired multi-view images are first turned into 3D point clouds. The 3D point cloud reconstruction includes sparse reconstruction based on SFM (structure from motion) algorithm and dense reconstruction based on the MVS algorithm. Here, batch reconstruction of acquired plants was realized by integrating open-source library OpenMVG and OpenMVS (Locher et al., 2016; Moulon et al., 2016). Vertices in the reconstructed point cloud involve both space coordinates and colors. Before denoising and sampling, the point number of a single scene should be >30 million (Figure 4A). In addition, a commercial software ContextCapture (Bentley, v.4.4.9), was used for 3D point cloud reconstruction to estimate the quality and usability of acquired image in commercial software.

#### Point cloud calibration and shoot segmentation

Unlike the sensors such as LiDAR or depth camera, the size of point clouds generated based on MVS technology is affected by plant size, shooting position, camera angle, and camera configuration. Besides, it has a different global coordinate system and scale of point cloud. Therefore, it is necessary to calibrate generated point cloud and correct the positive direction of the point cloud to transform the point cloud to their real size with the *XOY*-plane as the reference plane and the *Z*-axis as the positive direction. Furthermore, plant shoots should be segmented to facilitate further phenotype extraction. Therefore, the reconstructed point cloud should undergo calibration and segmentation.

##### Coarse-point-cloud-cropping

The center part of the reconstructed scene with the target shoot should be roughly segmented, and the initial point cloud should be down-sampled to improve computational efficiency. In center part cropping, the central point of point cloud was used as center of a cylinder. The point cloud was projected on the *XOY*-plane, and two-third diameter of the circumcircle of the projected point cloud, which empirically covers the wheat points and excludes the cloth fence points, is used as the diameter of the cylinder to construct the cropping cylinder and remove the point cloud outside the cylinder (Figure 4B). The points lower than 10% height in the *Z*-axis direction were removed to obtain the point cloud (Figure 4C). The number of remaining points after cropping was about 0.8~2 million. The point cloud was further simplified using random sampling to obtain 10% points (Figure 4D), which is promising for phenotype estimation accuracy and efficiency.

##### Calibration

The point cloud size was calibrated using plane attribute and circle radius of the marker plate set near the target plant in the scene. The color threshold was used to segment the marker plate since the surface color of the marker plat was known (red). The hue, saturation, and lightness (HSL) space can intuitively present the hue, saturation, and lightness compared with red, green, and blue (RBG) space. Therefore, the HSL color space was used to extract the point cloud of marker plate. The conversion from RGB to HSL space is shown in Equation (1).


(1) $$\begin{array}{l} h=\{\begin{array}{ll} 0^{{}^{\circ}} & \text{if }max=min\\ 60^{{}^{\circ}}\times \frac{g-b}{max+min}+0^{{}^{\circ}} & \text{if }max=r\text{ and }g\geq b\\ 60^{{}^{\circ}}\times \frac{g-b}{max+min}+360^{{}^{\circ}} & \text{if }max=r\text{ and }g<b\\ 60^{{}^{\circ}}\times \frac{b-r}{max+min}+120^{{}^{\circ}} & \text{if }max=g\\ 60^{{}^{\circ}}\times \frac{r-g}{max+min}+240^{{}^{\circ}} & \text{if }max=b \end{array}\\ l=(max+min)/2\\ s=\{\begin{array}{ll} 0 & \text{if }(max=min)\text{ or }(l=0)\\ \frac{max-min}{2-max-min} & \text{if }l\geq 0.5\\ \frac{max-min}{max+min} & \text{if }l<0.5 \end{array} \end{array}$$

where *max* = max(*r, g, b*), *min* = min(*r, g, b*), and *r, g, b* ∈ [0, 1]. The point was regarded as a point of the marker plate if the color of a point satisfied *h* > 340, *s* > 0.6, and *l* > 0.3 (Figure 4E). The boundary points were detected, and the circumcircle of the marker plate was estimated (Figure 4E). The diameter ratio between the estimated circumcircle and the real size of the marker plate was used as the scaling factor of the shoot point cloud.

The scaling accuracy of the marked plate point cloud after calibration was evaluated by comparing it with manual measurements obtained by measuring the diameter of the marker plate point cloud after scaling using an open-source software CloudCompare (http://www.cloudcompare.org/). Each measurement was repeated 5 times and averaged to eliminate the manual error. The error between the estimated and actual diameter was quantitatively evaluated using indicator mean absolute percentage error (MAPE) as shown in Equation (2), where ${\tilde{d}}_{i}$ is the estimated diameter from the point cloud, and *d*_*i*_ is the real size of the plate (6 cm).


(2) $$\begin{array}{l} MAPE=100\%\frac{1}{n}\overset{n}{\underset{i=1}{\sum }}\frac{\left|{\tilde{d}}_{i}-d_{i}\right|}{d_{i}} \end{array}$$

##### Shoot-point-cloud-cropping

The spatial coordinates of the marker plate were extracted. It was easy to calculate the height of the upper edge of the pot since the height of the marker plate and the height of pots were known. The points below the upper edge of the pot were removed, and a plant point cloud was obtained (Figure 4F). Statistical denoising (Rusu and Cousins, 2011) removed small point clusters in the retained points to realize plant point cloud denoising (Figure 4G).

#### The 3D phenotype extraction

The extracted and calibrated point clouds were used to estimate the 3D phenotyping parameters of plant shoots, including plant height, projected area, multi-layer projected area, leaf area, convex volume, and compactness.

##### Plant-height

The height difference between the maximum and minimum value of the point cloud on the Z-axis was considered the plant height.

##### Projected-area-and-multi-layer-projected-area

**Figure 5:**
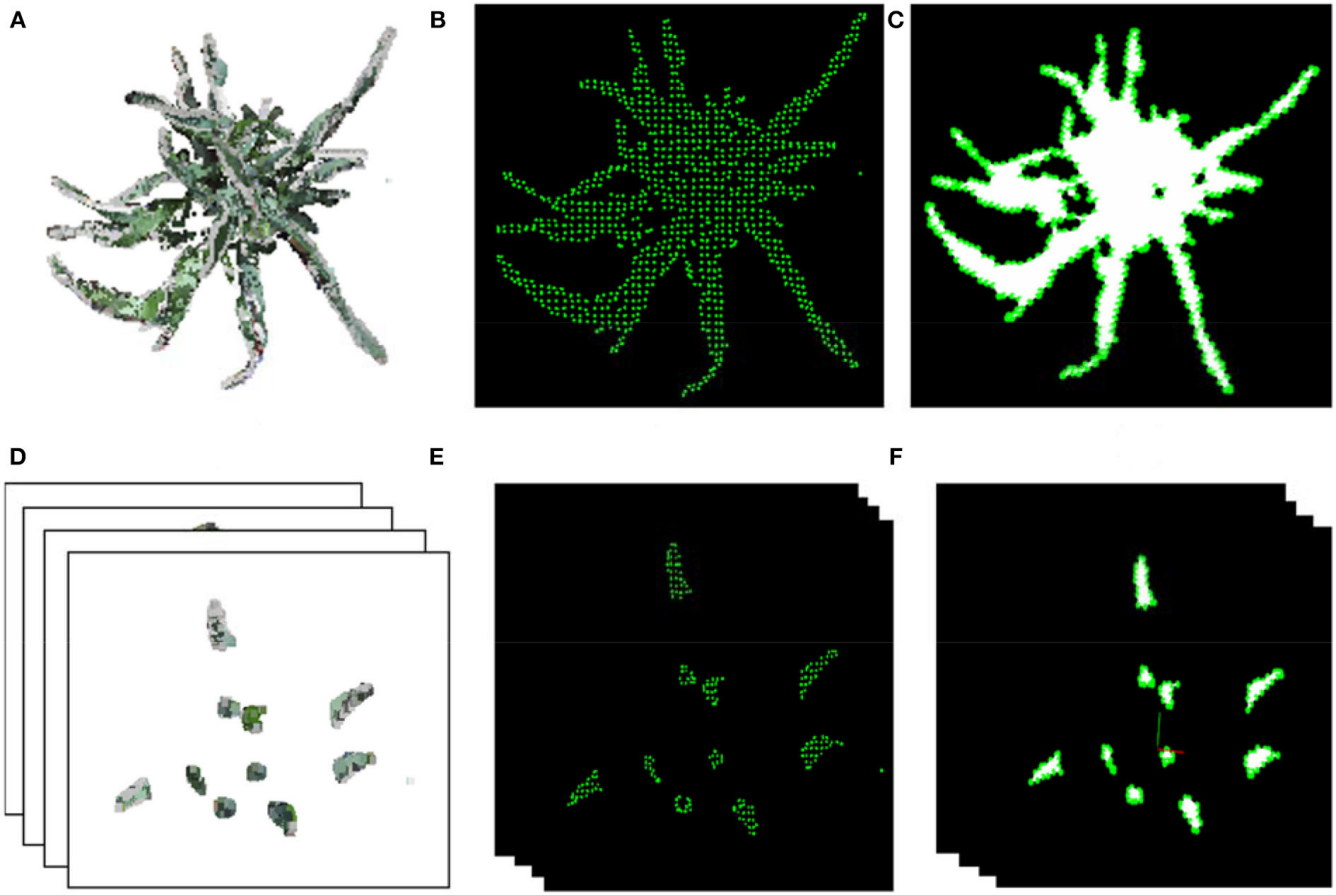
The projected area and multi-layer projected area. **(A,D)** Projected point cloud on *XOY*-plane of a shoot and layers. **(B,E)** Sparsely sampled points in the *XOY*-plane of the shoot and corresponding layer in **(A,D). (C,F)** Generated 2D triangle mesh of points in **(B,E)** for area estimation.

The shoot point cloud was first projected on the *XOY*-plane (Figure 5A), and the projected point cloud was then sparsely sampled using voxel filtering (Figure 5B). The greedy triangulation was used to generate mesh from the sparsely sampled points (Figure 5C). Finally, the sum area of the triangulation mesh was considered the projected area of the input shoot. The shoot point cloud was divided into several segments (equal height) for multi-layer projected area, and the multi-layer projected area was calculated as the projected area (Figures 5D–F).

##### Leaf-area

**Figure 6:**
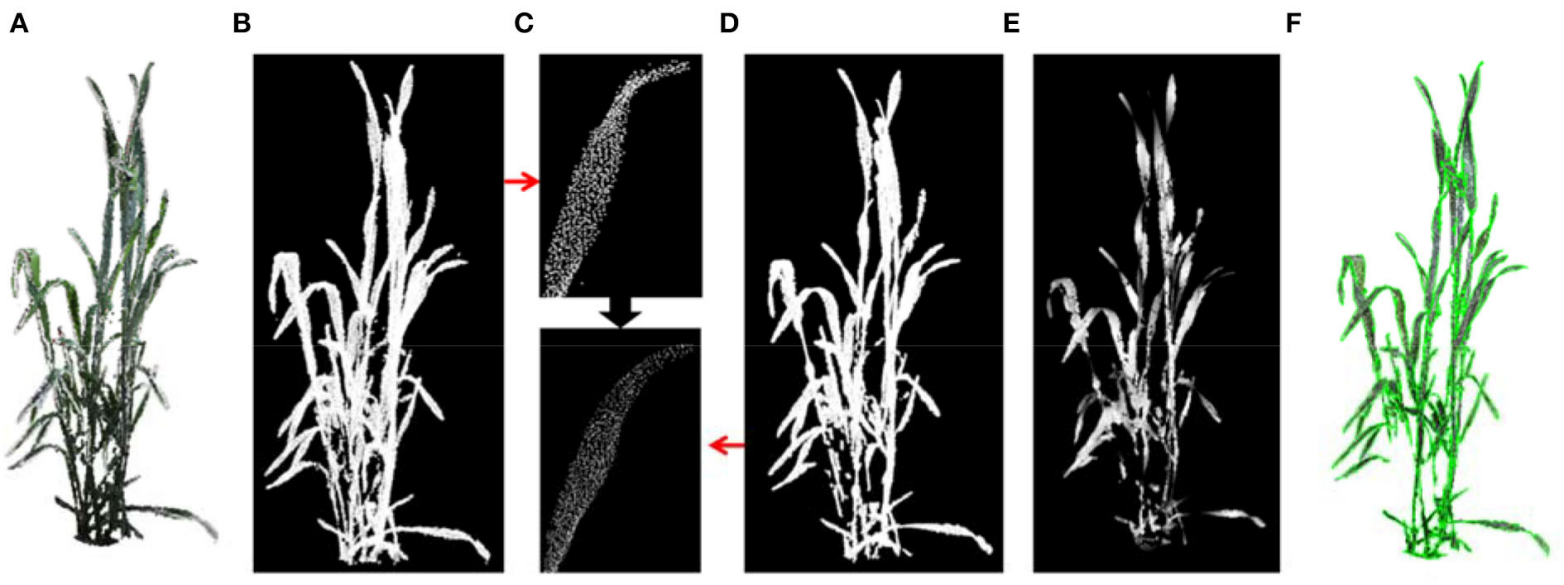
The leaf area estimation. **(A)** Wheat shoot point cloud. **(B)** Down sampled point cloud. **(C)** A leaf before and after point cloud smoothing. **(D)** Point cloud of a shoot after smoothing. **(E)** Triangulated mesh from the point cloud **(D)**. **(F)** Merged visualization of generated mesh and original colored point cloud.

Wheat has thin stems, narrow and long leaves with flat and smooth surface features. As a result, meshing the whole plant point cloud can effectively retain the leaf while removing stem points to calculate leaf area. First, plant point cloud (Figure 6A) was down sampled using the voxel grid method to improve the next-stage computational efficiency and ensure the uniformity of point cloud density (Figure 6B). The point cloud was then smoothened using the moving least square method (Alexa et al., 2003) (Figure 6C). Practically, the fitting polynomial in the smoothing procedure was set at 3 to maintain the bending and twisting stage of the blade (Figures 6C,D). Furthermore, the greedy triangulation was used to generate mesh from the smoothened point cloud. Most stem points were removed after smoothing and meshing (Figure 6E). Finally, the sum of all triangular facet areas was calculated as the shoot leaf area. The merged visualization of the generated mesh and original colored point cloud is shown in Figure 6F.

##### Shoot-convex-volume-and-compactness

**Figure 7:**
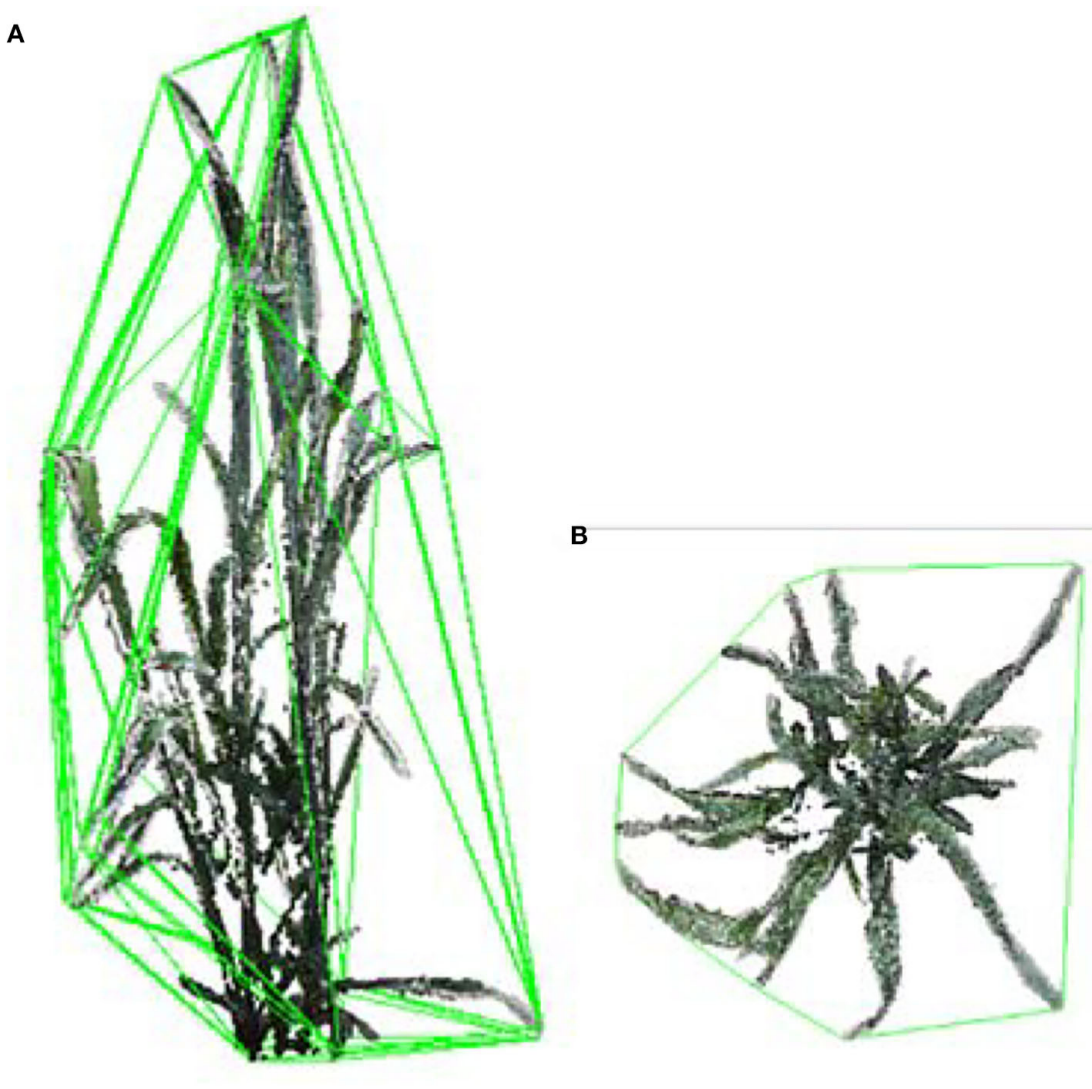
The shoot convex volume and compactness calculation. **(A)** Convex hull volume of a wheat shoot. **(B)** Convex hull area of projected plant points on a plane.

The shoot convex volume was estimated by calculating the convex hull of plant point cloud (Figure 7A). The compactness of a shoot was considered as the ratio of the projected area (Figure 7B) to the convex hull area. The shoot was more compact when the compactness was larger.

## Results

### Performance and applicability of MVS-Pheno V2 platform

The MVS-Pheno V2 is an improvement of the MVS-Pheno V1 based on hardware structure and software system for high-precision phenotype acquisition of small plant individuals or plant organs. The platform parameters and performance comparison of the two version platforms are shown in Table 2. The volume and height of V2 platform after disassembling are about quarter and one-third of those of the V1 version, respectively; thus, very portable. The light supplement and wind-shield modules were added to the V2 platform to reduce the disturbance of reconstructed data by inconsistent light and wind, but the overall cost did not increase. Moreover, the transportation structure was added to the V2 platform to make the multi-view data acquisition process more automatic. The V2 platform also systematically integrates with an open-source MVS reconstruction system, a robust calibration system, automatic point cloud phenotype calculation module, and network module. Consequently, a pipeline for automatic calculation of 3D plant phenotyping parameters from multi-view images was constructed.

**Table 2:** The MVS-Pheno platform parameters and performance comparison between V1 and V2.

**MVS-Pheno platform version**	**V1**	**V2**
Platform size after setup (m)	4.0 × 4.0 × 3.0	2.0 × 1.5 × 1.5
Platform size after disassembling (m)	2.0 × 1.0 × 1.0	1.5 × 1.2 × 0.3
Weight (kg)	180	60
Light supplement module	×	√
Windshield module	×	√
Cost (USD)	7,560	7,210
Measuring plant height range	[0.5, 2.5]	[0.1, 1.0]
Measuring plant width range	[0.3, 1.8]	[0.1, 0.8]
Automatic data acquisition	×	√
MVS-Reconstruction	Commercial software	Open-source system integration
Automatic post-processing	×	√
Applicable plants	Maize, Sorghum, cotton, hanging tomato, etc.	Wheat, rice, leafy vegetables, plants at the seeding stage, plant organs.

The data acquisition and processing efficiency (wheat shoot) using the MVS-Pheno V2 platform is shown in Table 3. The multi-view images of each shoot were acquired within 60 s, excluding the shoot incoming and outgoing time. The time interval of cameras was 2 s. The rotating speed is adjustable with a range from 0.5 to 2 rpm (rotations per min), and was set to 1 rpm in practice. The data was then automatically uploaded to a high-performance server for subsequent batch data processing. A point cloud reconstruction was the most time-consuming procedure. The point cloud pre-processing (point cloud calibration and denoising) and phenotype extraction were completed in seconds. The number of points obtained after denoising and down-sampling was within 20,000 for one camera and 80,000 for double camera acquired images, ensuring the point cloud quality; thus, improving phenotype extraction efficiency in later stages.

**Table 3:** Data acquisition and processing efficiency using MVS-Pheno V2 platform.

**Date**	**Camera number**	**Shoot height (cm)**	***t*_1_ (s)**	***n*_1_**	***t*_2_ (s)**	***n*_2_**	***t*_3_ (s)**	***n*_3_**	***t*_4_ (s)**
2021/03/23	1	<30	60	28	1,260	5.6 million	3	17,200	5
2021/04/02									
2021/04/13	2	<80	60	56	3,360	9.8 million	4	78,811	8
2021/04/19									

*t_1_: time of data acquisition, t_2_: time of MVS reconstruction, t_3_: time of pre-processing, indicating time cost for point cloud calibration and shoot segmentation, t_4_: time of phenotype estimation in section 3.5.3, n_1_: multi-view image number, n_2_: point number of the reconstructed scene, n_3_: shoot point number.

**Figure 8:**
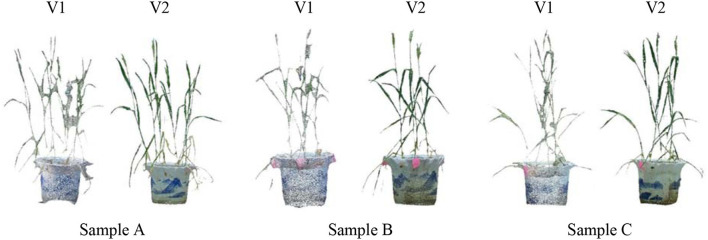
Visualized comparison of reconstructed point clouds from the acquired multi-view images using two version MVS-Pheno platforms of randomly selected potted wheat plants. For each subfigure, the left and right ones were reconstructed from images acquired using MVS-Pheno V1 and V2, respectively.

Three potted wheat plants were randomly selected and multi-view images were acquired using MVS-Pheno V1 and V2, respectively, to intuitively demonstrate the improvement of MVS-Pheno platform. Figure 8 shows the visualized comparison results of the reconstructed point clouds. The MVS-Pheno V2 platform significantly improves the point clouds quality. Point clouds reconstructed using V1 platform are not clear in leaf edges, and adjacent organs are connected. Points missing can be observed in many organs. However, point clouds reconstructed using V2 platform was relatively complete with clear edges, and adjacent organs were accurately connected. The comparison demonstrates the improvement of the platform was necessary and effective, especially for complex and small-size plants.

### Point cloud calibration results

**Figure 9:**
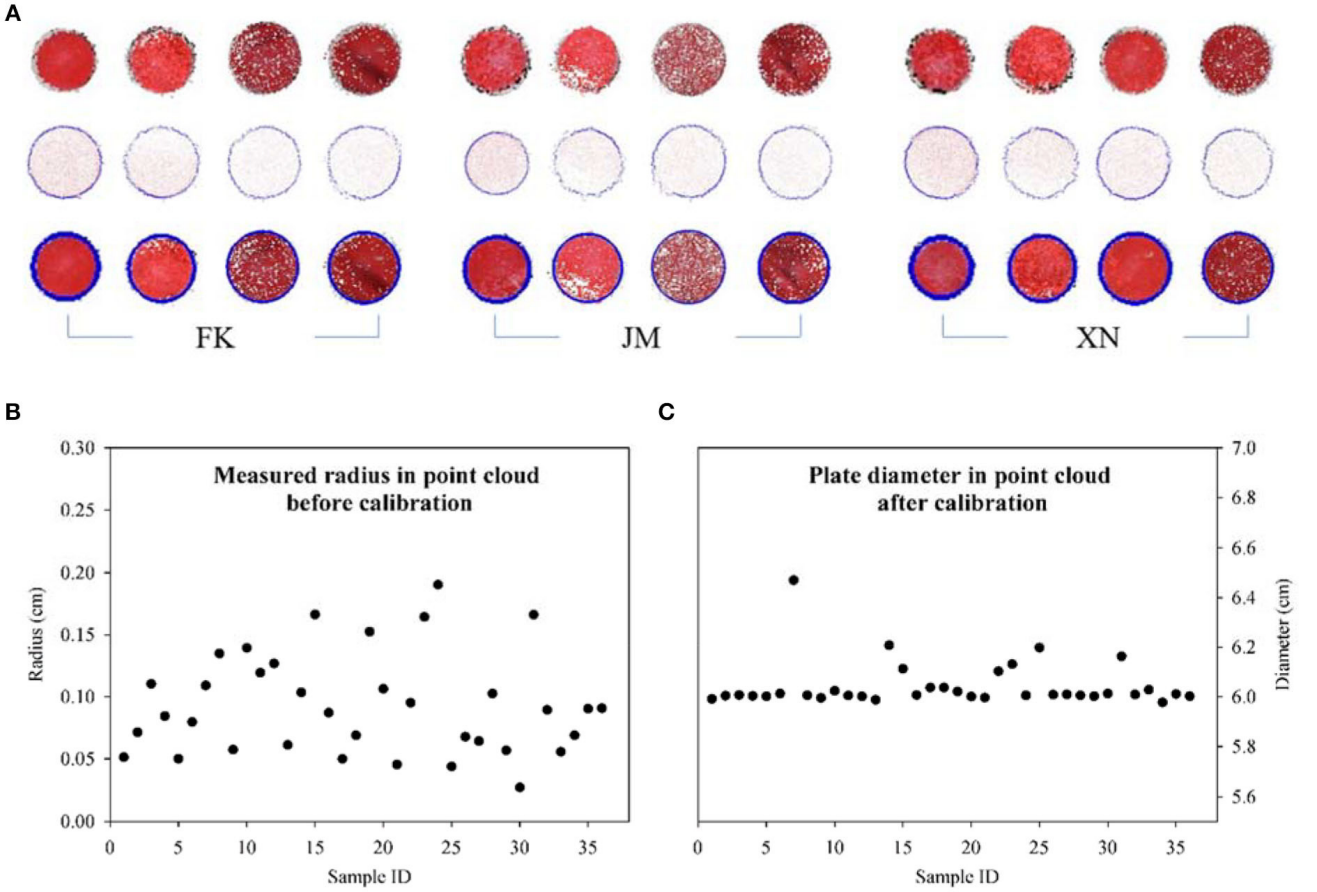
Visualization results and error comparison of marker plate measurements. **(A)** Identified marker plate and radius estimation. Three rows represent segmented marker plate points, extracted boundary points, and fitted circumcircle, respectively. **(B)** The measured radius of marker plate before point cloud calibration. **(C)** Measured diameter of marker plate after point cloud calibration. Errors of calibrated diameters occur because the marker plate being partly covered by wheat leaves.

Automatic segmentation, extraction, and measurement marker plate in the reconstructed point cloud were realized when the plate was not in contact with the target plant. Marker plate point cloud was extracted from the reconstructed scene, and the circumcircle was estimated using the cultivar wheat plant sample scenes at different growth stages (Figure 9A). Even if the equipment and sensor positions were fixed, the generated point clouds were not reconstructed in equal scales under different acquisition scenarios. The manually measured radius conducted in CloudCompare of the marker plate point cloud under different plant scenes ranged from 0.02 cm to 0.2 cm, while the actual radius of the marker plate was 3 cm (Figure 9B). The 36 samples had different measured values of each marker plate, explaining why scale calibration was needed for each multi-view reconstruction scene, instead of just once calibration before measurement. Furthermore, the radius of the marker plate in each scene after scale calibration was also manually measured to evaluate the reliability of the scale calibration method. The measurement results are shown in Figure 9C. Except for sample No. 7 (FK-2 obtained on 13 April 2021), where the measured value discrepancies were considerable due to the marker plate being partly covered by wheat leaves during multi-view image capture, the difference between estimated and measured values for the other samples was minimal. The mean absolute percentage error (MAPE) and maximum error were 0.585 and 3.3%, respectively, indicating that the scale calibration method was accurate.

### Point cloud reconstruction results

#### Point cloud visualization

**Figure 10:**
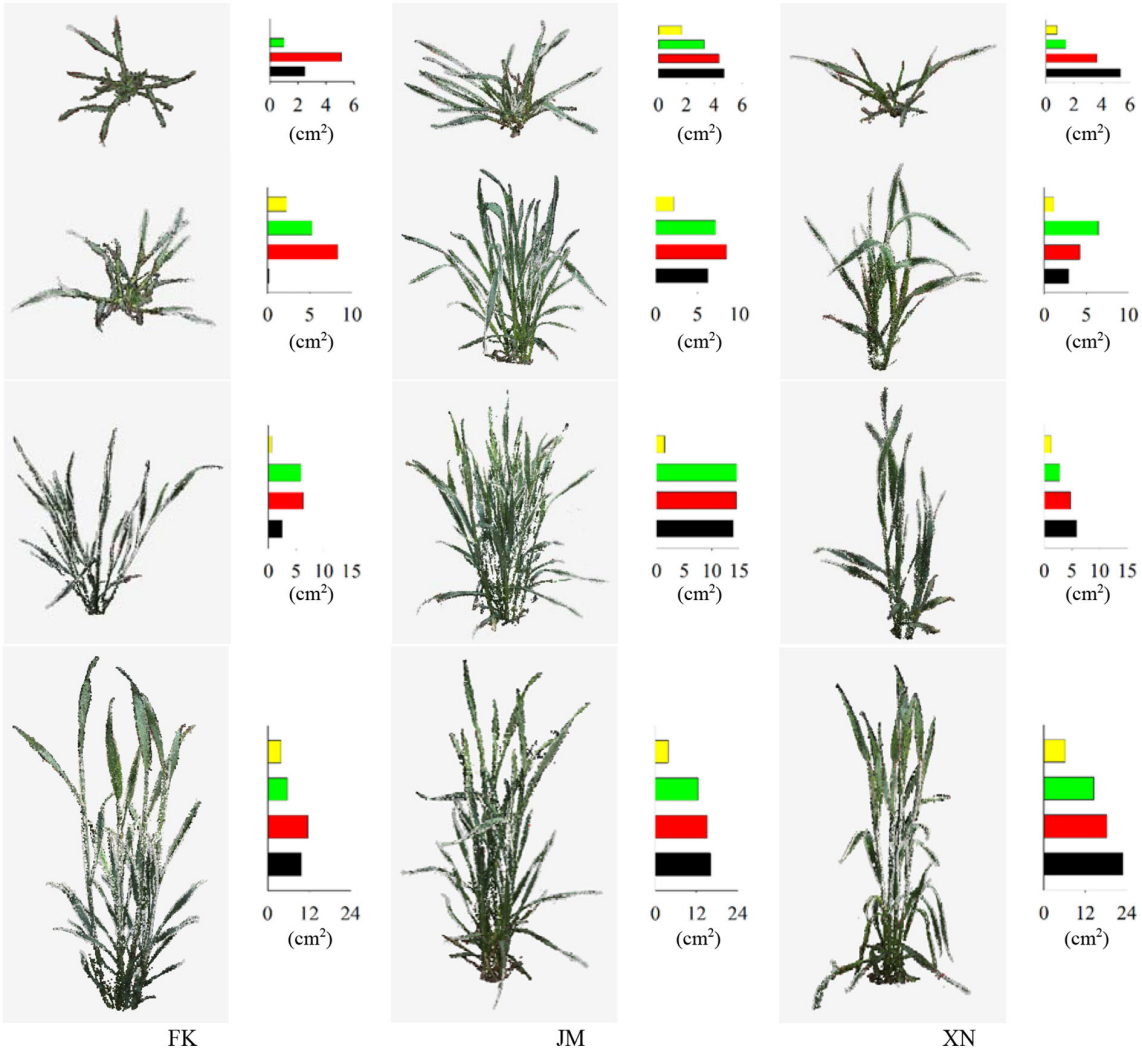
Point cloud visualization of reconstructed wheat shoots of three cultivars at four growth stages. Bar charts represent projected leaf area (cm^2^) in four layers. The colors, black to yellow, indicate layered projected area from bottom-to-top layers.

The point cloud visualization of the reconstructed wheat shoots of the three cultivars at four growth stages is shown in Figure 10. The reconstructed wheat shoot point cloud at different stages was satisfactory, as discussed in the following results: Tillers of all shoots were completely reconstructed and had clear edges. The edge of the leaf points was not missing, the leaf tips were retained, and no holes exist (a few holes were observed in leaves at the lower part of the internal tillers) from the small leaf with a width of 0.5 cm and a length of 5 cm at the seedling stage, to big leaf with a width of 2.5 cm and a length of 25 cm at the booting stage. The reconstruction results for wheat plants with more tillers and compact shoot architecture which were relatively difficult to reconstruct, such as cultivar JM (Figure 10) were also satisfactory.

**Figure 11:**
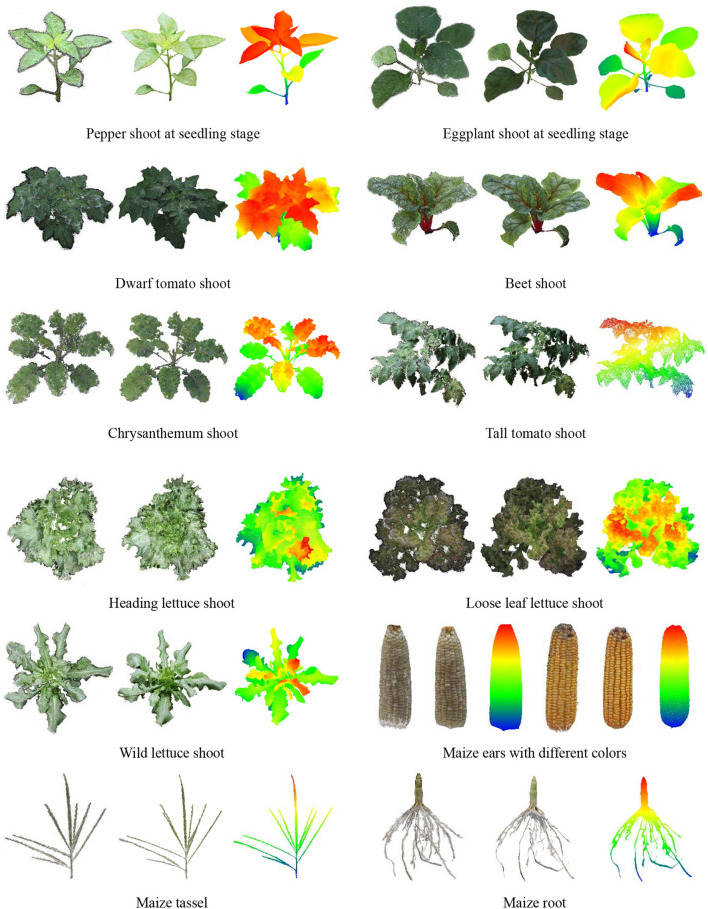
Point cloud visualization of other plants and plant organs. The left ones were reconstructed using open-source 3D reconstruction program integrated into MVS-Pheno V2. The middle ones were reconstructed using ContextCapture software. The left and middle point clouds were visualized using vertex colors for each group, and the right ones were visualized using color differences along with the height direction of the middle point clouds.

Seedling plants, leafy plants with complex leaves, and plant organs were used to test the performance of the platform in other types of plants and other MVS reconstruction software. The MVS-Pheno V2 platform was used to obtain multi-view images. The open-source 3D reconstruction program integrated into MVS-Pheno V2 and ContextCapture software were used for 3D point cloud reconstruction. The reconstructed point clouds are shown in Figure 11. Good reconstruction point clouds with realistic colors were obtained, demonstrating that the platform was also applicable in other plants as long as the size of the plants is suitable. Moreover, the multi-view images acquired using this platform are available for other MVS reconstruction algorithms and software. The point clouds reconstructed using ContextCapture were optimized with fewer noise and seemed relatively clean. The point clouds obtained using open-source algorithms were with noises, but were relatively complete.

#### Point cloud resolution and accuracy

**Figure 12:**
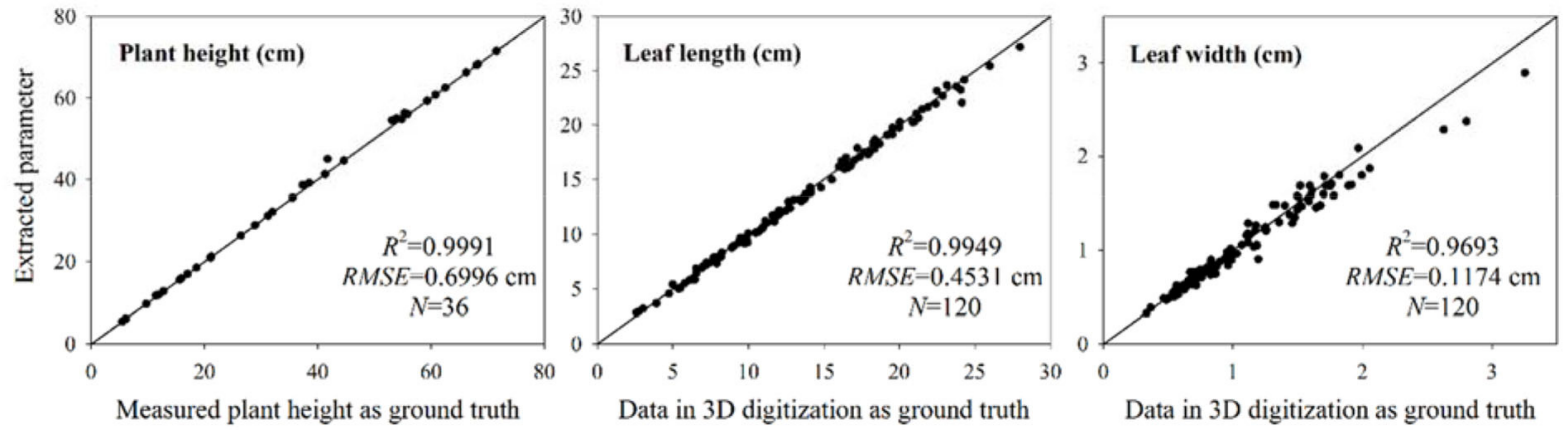
Accuracy evaluation of the extracted plant height, leaf length, and leaf width. The extracted plant height was compared with the measured values. The measured leaf length and width values from the reconstructed point clouds and the estimated values from the 3D digitization data were compared. The estimated values were regarded as the basis for comparison.

The accuracy of the reconstructed point cloud was quantitatively evaluated by manually measuring plant height, leaf length, and width of wheat shoots. The plant height is an essential indicator of the scaling accuracy of reconstructed point clouds. The estimated plant heights of 36 sample shoots were compared with the manual measurement (Figure 12). The *R*^2^ and root mean squared error (RMSE) were 0.9991 and 0.6996 cm, respectively. The plant height error occurred in several shoots due to marker plate scaling error (Figure 9C). However, the overall performance of the extracted plant height was satisfactory. A 3D digitization data acquired using a 3D digitizer was used to estimate leaf length and width, which can be regarded as the ground truth in comparison (Wang et al., 2019). Due to the difficulty in realizing automatic leaf segmentation and recognition, corresponding tillers of the acquired digitizer data were found from the point cloud reconstructed using the platform, and the CloudCompare software was used to measure the maximum leaf length and width manually. Leaf width was measured at the widest part of each leaf. A total of 120 leaves randomly selected from upper, middle, and bottom positions among 36 sample plants were measured. The comparison results between the extracted values (measured manually using software) from the point clouds and estimated values from the 3D digitization data are shown in Figure 12. The *R*^2^ of the leaf length and width were 0.9949 and 0.9693, respectively. The RMSE of the leaf length and width were 0.4531 cm and 0.1174 cm, respectively. These results demonstrated that the reconstructed wheat plant using MVS-Pheno V2 platform had high accuracy and could retain leaf tip and edge features.

### Extracted phenotype analysis of wheat shoots

**Figure 13:**
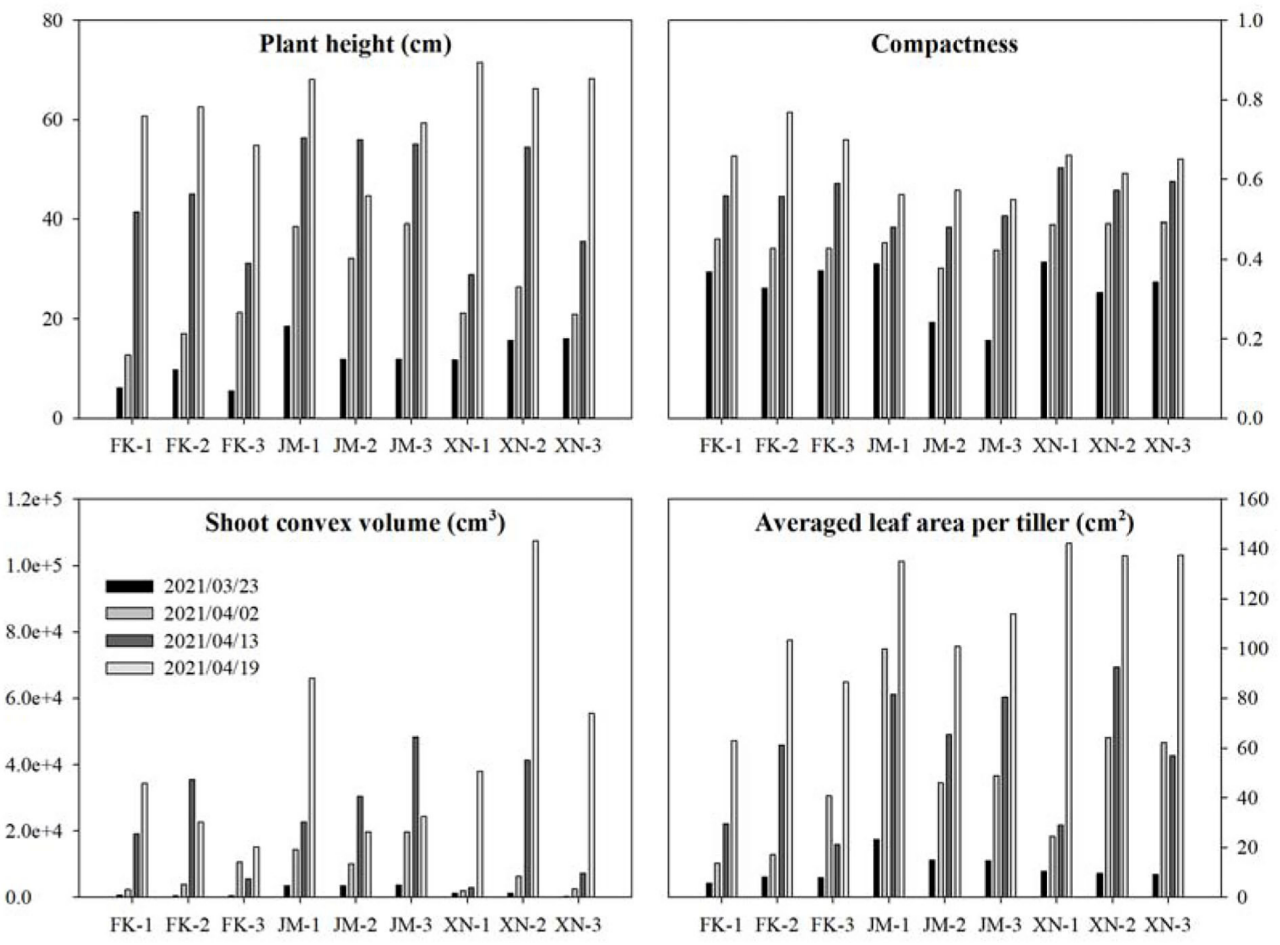
Estimated phenotypes of three wheat cultivars at four growth stages. Each cultivar had three samples. The estimated phenotypes include plant height, compactness, shoot convex volume, and averaged leaf area per tiller.

Phenotypes of 36 sample shoots were calculated (Figure 13). The FK had the shortest plant height at each growth stage. The JM had the longest plant height on 13 April 2021, while XN had the longest on 19 April 2021 (Figure 13). The compactness of each cultivar gradually increased with the growth process. Cultivar JM had the smallest compactness in the late growth stage, indicating that JM had a looser shoot architecture than other cultivars. The averaged leaf area was calculated based on the tiller numbers of each shoot to compare the tiller phenotypes (Table 1). Cultivar XN had the largest averaged leaf area while FN had the smallest averaged leaf area per tiller on 19 April 2021. These results indicated that XN had a larger leaf area than other cultivars in each tiller. Shoot convex volume and averaged leaf area per tiller increased with the plant growth. Figure 13 demonstrates that the platform is able to capture the phenotype differences among cultivars and individual plants.

Each cultivar plants in different growth stages were not continuously measured using specific shoots because samples shoots were destructively transplanted from the field, leading to unsustainable increasing data in the averaged leaf area per tiller, such as FK-3, JM-1, and XN-3. The projected leaf area gradually increased with the growth process (Figure 10). The second layer was the largest, the first layer was close to the third layer, and the fourth layer was the smallest (from bottom to top). The FK had the smallest projected leaf area.

## Discussion

### Hardware improvement of MVS-Pheno platform

The miniaturized MVS-Pheno V2 platform was an improvement of the MVS-Pheno V1 (Wu et al., 2020) for small plants. The improvements were as follows: (1) Wireless communication and control were realized in the V2 platform; thus, signal and data cables in the V1 platform were removed, avoiding complex cable winding and improving the overall performance. (2) The V2 platform had the top mounted turntable structure for allowing complete separation of turntable and plants to be measured. As a result, the plants maintain the static state, avoiding movement during the collection process; thus, reducing the noise of the reconstructed point cloud, and the reconstructed point cloud can retain fine features such as leaf tip and edge. Besides, the change of hardware size reduces the space required by the platform. (3) Better light source control was realized in the V2 platform by setting up enclosure structure and light supplement module, ensuring consistent and even lights; thus, obtaining high-quality images. (4) The V2 platform had a suitable image background compared with the V1 platform. The enclosure cloth filters out the disordered background. It effectively prevents the moving background, such as the dynamic noise caused by people walking within the camera field of view during image acquisition. (5) The V2 platform had a more suitable calibration structure; thus, enhancing accuracy and robustness. In summary, the four elements of the MVS reconstruction system include even illumination, clear background, static plant, and sufficient image overlap. The MVS-Pheno V2 provides systematic a design based on these four aspects to ensure the high-resolution and high-precision acquisition of plant multi-view images.

Notably, the turntable adopts precision gear transportation to improve its rotation stability. Therefore, the V2 platform cannot be easily disassembled by non-professionals, reducing its portability.

### Comparison with “camera to plant” methods and other types of 3D sensors

High-throughput 3D phenotyping of short plants is in high demand, and the “camera to plant” mode is commonly considered an effective and low-cost solution. Despite many efforts being made to achieve multi-view image acquisition (Rose et al., 2015; Hui et al., 2018), most of these methods are not automatic. The studies describing “camera to plant” have reported improved data acquisition efficiency. However, the hardware described in these studies seems to be prototype, and robustness and stability of these devices might not reliable (Nguyen et al., 2016; Cao et al., 2019). Besides the MVS-Pheno V1 platform (Wu et al., 2020), automatic control and data transformation were not considered in these studies. In contrast, MVS-Pheno V2 is highly automatic in data acquisition, and wireless control and data transformation are also involved in the system. Imaging environment, including light and airflow, are important factors for MVS-based 3D phenotyping. Although the imaging background and wind shelter were taken into account in Gao's research (Gao et al., 2021), the scenario was simply built to meet the basic demands of data acquisition. The MVS-Pheno V2 platform has light supplement and windshield modules, providing promising imaging environment.

The MVS-Pheno V2 platform was designed for low plant shoots; thus, the range was smaller than most 3D sensors. Because the point clouds were reconstructed using high-resolution images, the resolution and accuracy were satisfactory for plant phenotyping demands, and were better than Kinect and low-resolution LiDARs. The MVS-Pheno V2 platform was automatic; thus, easy for users to acquire and process data. Comparatively, other types of 3D sensors generally need to move around a target plant manually. The cost of the MVS-Pheno V2 platform was nearly 8,500 dollars, which was cost-effective compared with high-resolution 3D scanners, such as FARO Focus.

### Improvement of data processing system

The data processing system of the MVS-Pheno V2 platform systematically integrates open-source libraries and realizes batch reconstruction of point clouds using multi-view images. Moreover, automatic point cloud calibration and phenotype extraction are also involved in the system. Consequently, an automatic data processing pipeline was established, which is essential for handling big data in plant phenomics (Zhang et al., 2017).

Point clouds generated using MVS reconstruction technology are unequally scaled under different data acquisition scenarios, even if the equipment and sensor positions remain unchanged and the positive direction of the reconstructed point cloud are inconsistent. Therefore, besides calibrating the point cloud scale, the calibration system should also correct the positive direction. Herein, the designed calibration system was robust and had satisfactory reconstruction accuracy, effectively ensuring automation for post-data processing. The extracted 3D phenotypes described in this study can also be achieved using existing methods. They were listed here to promise the integrity of MVS-Pheno V2, including hardware design and automatic data processing system. Unlike the data acquired using 3D digitizers (Zheng et al., 2022), the reconstructed point clouds of plants using MVS-Pheno V2 platform are unordered and without semantic information. Further point cloud feature extraction and analyzing algorithms should be developed to extract agronomy traits for next-stage applications.

### Platform applicability and future improvements

The MVS-Pheno V2 platform has a limited imaging range and is unsuitable for tall and large plants described in the V1 platform (Wu et al., 2020). However, The V2 platform is more suitable for lower plants with or without branches or tillers and can also be used for plant organs, such as maize tassels, ears, and roots. The reconstructed point clouds of plant shoots were used to estimate 3D phenotyping characteristics such as leaf area, layered projected area, volume, and compactness. The MVS-Pheno V2 platform provides a low-cost and high-throughput solution for the 3D phenotyping of individual plants. Shielding affects plants with too compact tillers or a small group of plant population. Besides, the V2 platform cannot effectively reconstruct internal and lower leaves. Recovery of the missing points is also difficult for data acquired using LiDAR.

However, the platform needs some improvements in the future. For instance, the hardware should be made lighter and easier to disassemble to make the platform easier to deploy and save labor costs. Point cloud segmentation (Miao et al., 2021) and phenotype extraction algorithms for specific plant species should be developed.

## Conclusion

A multi-view stereo reconstruction-based phenotyping platform MVS-Pheno V2 was developed for small plant shoots. The platform is composed of hardware and data processing system and can realize automatic and high-throughput data acquisition and phenotypes extraction. The hardware is a miniaturized equipment that needs small space to deploy. Controlled imaging condition is established to avoid light and wind affection and ensure image quality. Wireless communication and control were integrated to avoid cable twining. Data processing system includes 3D point cloud reconstruction using multi-view images, point cloud calibration that returned the point cloud to plant real size, and 3D phenotype extraction model. MVS-Pheno V2 is applicable for wheat, rice, leafy vegetables, plants at the seeding stage, and plant organs. The *R*^2^ was 0.9991, 0.9949, and 0.9693 for plant height, leaf length, and leaf width, respectively. The RMSE was 0.6996 cm, 0.4531 cm, and 0.1174 cm for plant height, leaf length, and leaf width. These results demonstrating the point cloud quality is satisfactory for wheat phenotypes measurement.

## Data availability statement

The original contributions presented in the study are included in the article/Supplementary material, further inquiries can be directed to the corresponding authors.

## Author contributions

Conceptualization: SW and XG. Data curation: XL, WZ, CZ, and ZX. Formal analysis, methodology, and writing—review and editing: SW and WW. Funding acquisition and supervision: LC and XG. Resources: XL. Software: SW and WG. Validation: SW and WZ. Writing—original draft: SW, WW, and XG. All authors contributed to the article and approved the submitted version.

## Funding

This work was partially supported by Construction of Collaborative Innovation Center of Beijing Academy of Agricultural and Forestry Sciences (KJCX201917), Science and Technology Innovation Special Construction Funded Program of Beijing Academy of Agriculture and Forestry Sciences (KJCX20210413), the National Natural Science Foundation of China (31871519 and 32071891), and China Agriculture Research System of MOF and MARA.

## Conflict of interest

The authors declare that the research was conducted in the absence of any commercial or financial relationships that could be construed as a potential conflict of interest.

## Publisher's note

All claims expressed in this article are solely those of the authors and do not necessarily represent those of their affiliated organizations, or those of the publisher, the editors and the reviewers. Any product that may be evaluated in this article, or claim that may be made by its manufacturer, is not guaranteed or endorsed by the publisher.

## Supplementary material

Supplementary Video 1 An animation illustrating the MVS-Pheno V2 platform.
